# Ultrasonography-based radiomics and computer-aided diagnosis in thyroid nodule management: performance comparison and clinical strategy optimization

**DOI:** 10.3389/fendo.2023.1140816

**Published:** 2023-05-12

**Authors:** Mengwen Xia, Fulong Song, Yongfeng Zhao, Yongzhi Xie, Yafei Wen, Ping Zhou

**Affiliations:** ^1^ Department of Ultrasonography, The Third Xiangya Hospital of Central South University, Changsha, China; ^2^ Department of Radiology, The Third Xiangya Hospital of Central South University, Changsha, China

**Keywords:** thyroid nodule, radiomics, computer-aided diagnosis, ultrasonography, risk assessment, prediction

## Abstract

**Objectives:**

To compare ultrasonography (US) feature-based radiomics and computer-aided diagnosis (CAD) models for predicting malignancy in thyroid nodules, and to evaluate their utility for thyroid nodule management.

**Methods:**

This prospective study included 262 thyroid nodules obtained between January 2022 and June 2022. All nodules previously underwent standardized US image acquisition, and the nature of the nodules was confirmed by the pathological results. The CAD model exploited two vertical US images of the thyroid nodule to differentiate the lesions. The least absolute shrinkage and operator algorithm (LASSO) was applied to choose radiomics features with excellent predictive properties for building a radiomics model. Ultimately, the area under the receiver operating characteristic curve (AUC) and calibration curves were assessed to compare diagnostic performance between the models. DeLong’s test was used to analyze the difference between groups. Both models were used to revise the American College of Radiology Thyroid Imaging Reporting and Data Systems (ACR TI-RADS) to provide biopsy recommendations, and their performance was compared with the original recommendations.

**Results:**

Of the 262 thyroid nodules, 157 were malignant, and the remaining 105 were benign. The diagnostic performance of radiomics, CAD, and ACR TI-RADS models had an AUC of 0.915 (95% confidence interval (CI): 0.881–0.947), 0.814 (95% CI: 0.766–0.863), and 0.849 (95% CI: 0.804–0.894), respectively. DeLong’s test showed a statistically significant between the AUC values of models (p < 0.05). Calibration curves showed good agreement in each model. When both models were applied to revise the ACR TI-RADS, our recommendations significantly improved the performance. The revised recommendations based on radiomics and CAD showed an increased sensitivity, accuracy, positive predictive value, and negative predictive value, and decreased unnecessary fine-needle aspiration rates. Furthermore, the radiomics model’s improvement scale was more pronounced (33.3–16.7% vs. 33.3–9.7%).

**Conclusion:**

The radiomics strategy and CAD system showed good diagnostic performance for discriminating thyroid nodules and could be used to optimize the ACR TI-RADS recommendation, which successfully reduces unnecessary biopsies, especially in the radiomics model.

## 1 Introduction

Thyroid nodules are common but often asymptomatic, and guidelines strongly recommend that all patients with known or suspected thyroid nodules undergo thyroid ultrasonography (US) with a survey of the cervical lymph nodes (1). With the widespread use of high-frequency US, the prevalence of thyroid nodules has been reported to be as high as 68%, with a higher proportion among populations with iodine deficiency and the elderly (2). The management of thyroid nodules has shown increased clinical importance due to the high incidence of nodules and soaring healthcare costs. However, operator-specific expertise and the inability to quantify image features frequently restrict the sensitivity and specificity of US diagnoses, which results in a lack of consistency and objectivity (3).

With the presentation and application of various risk-stratification systems, such as the Thyroid Imaging Reporting and Data System released by the American College of Radiology (ACR TI-RADS), standardized terminology has gradually been used to describe the appearance of thyroid nodules (4, 5). US has become a primary diagnostic tool used for the final classification of thyroid nodules and can help in decision-making regarding the use of fine-needle aspiration (FNA). However, due to the subjectivity, diversity, and overlapping risk features between the benign and malignant nodules, data on the interobserver agreement are weak (6).

Recent advances in technology have shown superiority in the differentiation of thyroid nodules. The use of computer-aided diagnosis (CAD) systems in the diagnosis of thyroid nodules seems to be a promising tool (7). Several artificial intelligence tools are commercially available that have received Food and Drug Association approval, such as S-detect, AmCAD-UT, Koios DS, and Medo Thyroid. Previous studies have shown that S-detect could provide second objective decision-making support *via* a semiautomated workflow in differentiating thyroid nodules from US images and reducing the rate of missed diagnoses (8–12). S-detect technology has been iterated several times, and can now identify calcification as an important clue. More recently, a new analysis method called radiomics, which is based on data science, quantifies the characteristics of lesions in medical images to extract a significant number of phenotypic features (13, 14). To our best knowledge, no published study has compared the accuracy of radiomics and CAD systems based on US features in the prediction of thyroid cancer for thyroid nodule management.

Therefore, this study aimed to prospectively evaluate the diagnostic efficiency of benign and malignant thyroid nodules using the US-based radiomics analysis method and CAD system while exploring their potential complementary role to ACR management recommendations.

## 2 Materials and methods

### 2.1 Patients

This study was approved by the ethical review committee of the Third Xiangya Hospital of Central South University, and written informed consent was obtained from all patients before they received examinations. Patients and data were collected prospectively randomized and double-blinded by a tertiary hospital.

**Figure 1:**
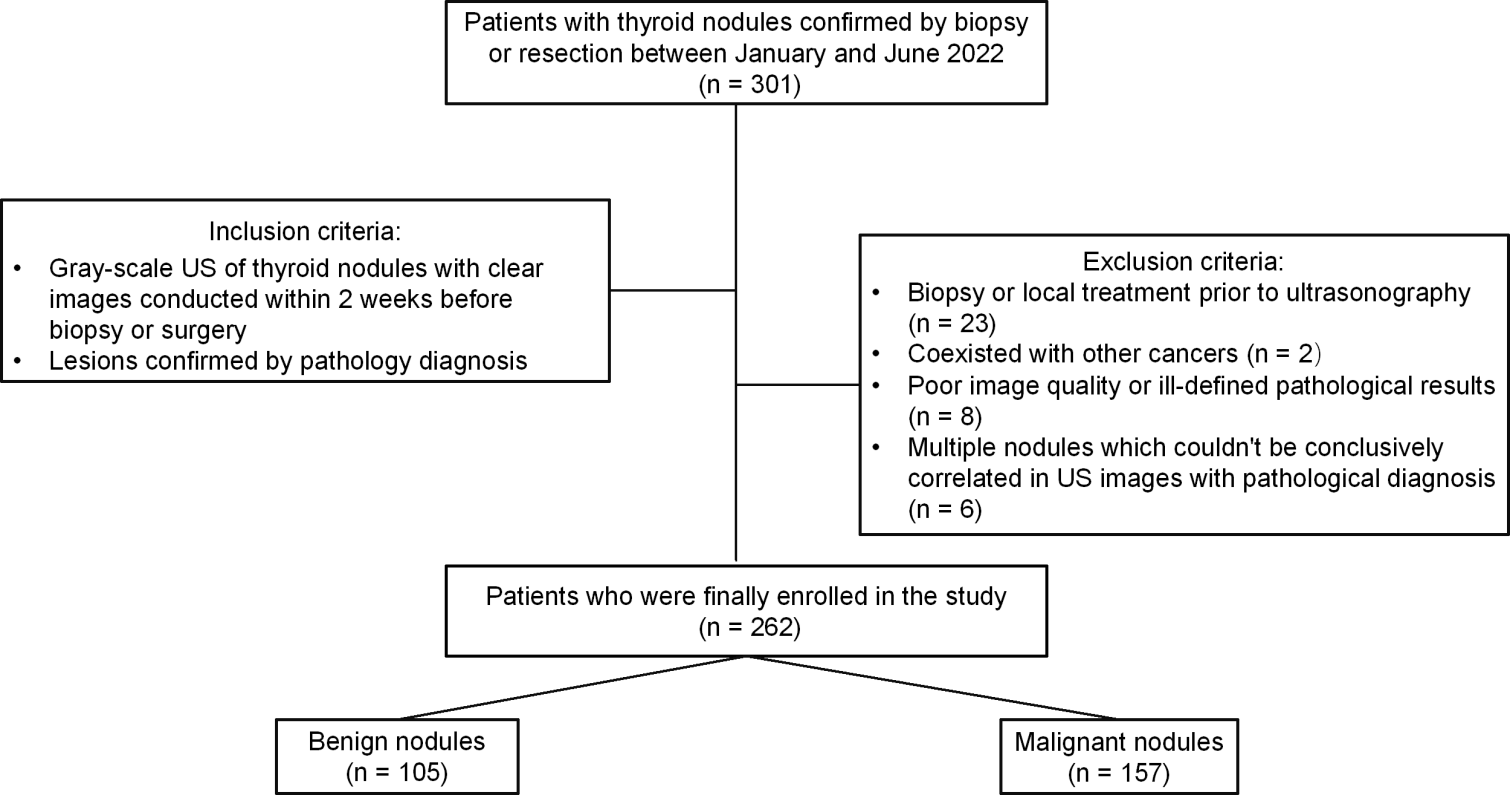
Flowchart of the study population.

A total of 301 thyroid nodules from 179 consecutive patients who had undergone regular preoperative gray-scale US imaging of thyroid nodules with clear images and had obtained a pathological diagnosis by FNA or surgical resection for lesions within 2 weeks were included at our institution between January 2022 and June 2022. Among the 301 thyroid nodules, 39 were excluded due to the following reasons (1): biopsy or local treatment before US (n =23) (2); other cancers (n = 2) (3); poor image quality or ill-defined pathological results (n = 8); and (4) multiple nodules could not be conclusively correlated in US images with pathological diagnosis (n = 6). Finally, this study included 148 patients in total with 262 thyroid nodules. **Figure 1** shows the flowchart of this study population. The final diagnosis was based on FNA or surgical histopathology.

### 2.2 Image acquisition and annotation

All US examinations were performed with Hera W10 (Samsung Medison) and a real-time CAD US system (S-Detect for Thyroid; Samsung Medison) using a 3–12 MHz linear probe. A senior trained radiologist with 25 years of experience in thyroid imaging independently performed all US examinations and numbered the nodules. Meanwhile, the thyroid nodule’s largest segment (longitudinal section) and its vertical section (transverse section) were measured for further image annotation.

#### 2.2.1 CAD image acquisition and annotation

**Figure 2:**
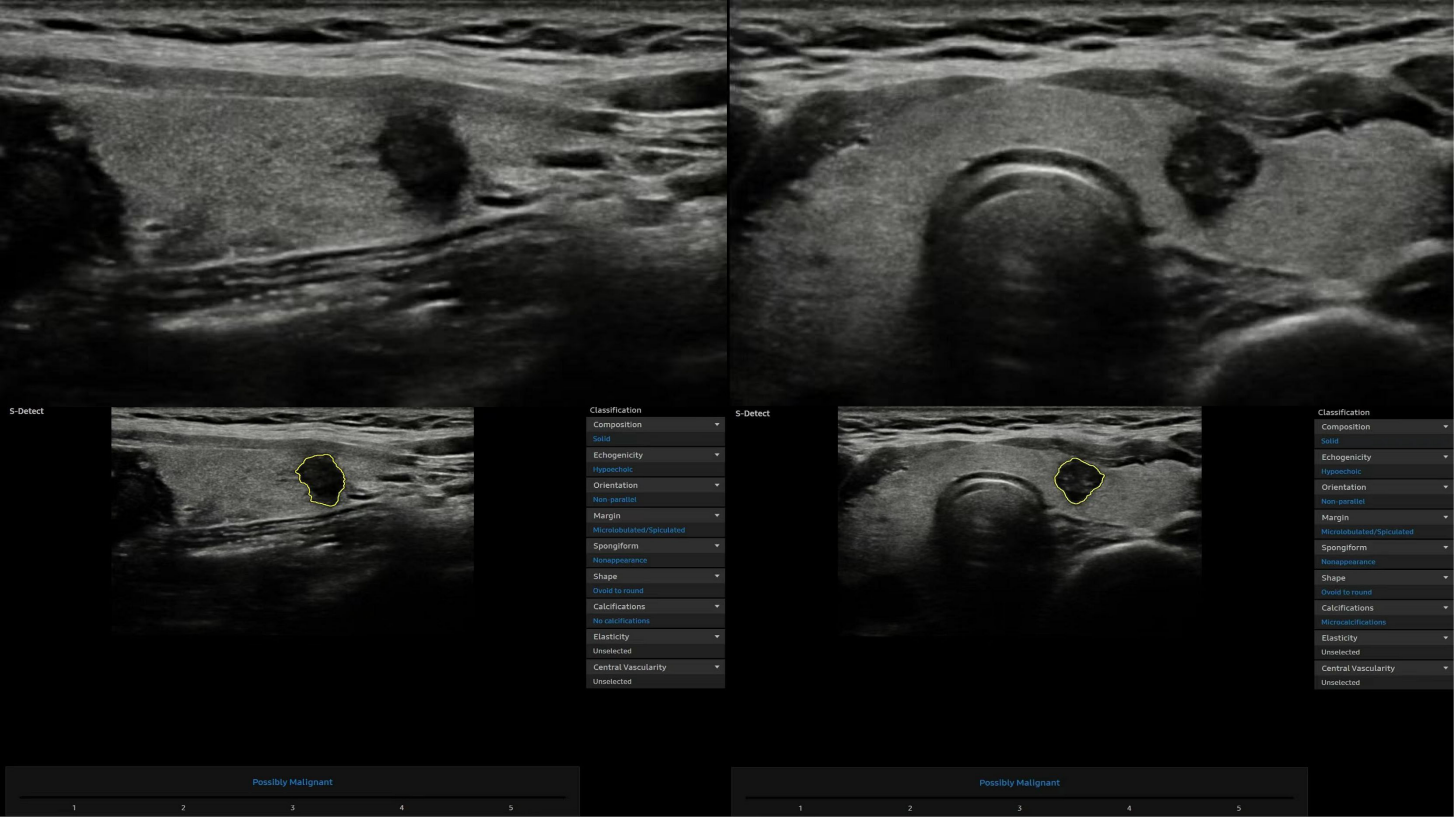
Representative thyroid nodule images were acquired with the computer-aided diagnosis (CAD) system.

The same sonologist analyzed CAD data with S-Detect on transverse and longitudinal sections immediately after image acquisition. After manually confirming the location of the lesion, the software automatically segmented the mass contours. The operator manually readjusted the outline if the contour border was dissatisfactory. The software analyzed US features of the lesion, including composition, echogenicity, orientation, margins, shape, calcifications, and spongiform appearance (8). Finally, S-Detect provided the diagnosis as “possibly benign” or “possibly malignant” in dichotomy form (**Figure 2**). In addition, if the assessments of two vertical sections were inconsistent, the malignant result was regarded as final.

#### 2.2.2 US image annotation

Sonograms were independently evaluated by an experienced senior thyroid imaging expert with 20 years of experience who was blinded to the pathological result according to ACR TI-RADS for composition, echogenicity, shape, margin, and echogenic foci (1). The reader independently assigned features of every nodule for the five ACR TI-RADS categories. Ultimately, all nodules had feature assignments, resulting in point assignments and corresponding TI-RADS risk classifications for each nodule.

### 2.3 ROI segmentation, feature extraction and selection

Without any knowledge of the other results, two radiologists (3 and 5 years of experience in thyroid imaging) independently performed the follow-up radiomics analysis. After normalizing the grayscale and voxels, the regions of interest (ROIs) were performed by a 3D Slicer (https://www.slicer.org/) (software version 5.0.2) to manually segment the thyroid nodules on the image in the transverse and longitudinal section. Operators were trained to segment ROIs before the study began. Intra-observer and inter-observer consistency was evaluated with a random cohort of 30 nodules segmented by one of the operators. One month after the first lesion segmentation, two operators completed the re-segmentation of this cohort image. The intraclass correlation coefficient (ICC) was used to assess the reproducibility and robustness of lesion segmentation and feature extraction.

A total of 837 candidate radiomics features were extracted from each ROI using the plug-in “PyRadiomics” package in 3D-Slicer, including features from first-order statistics, gray level co-occurrence matrix (GLCM), gray level dependence matrix (GLDM), gray level run length matrix (GLRLM), gray level size zone matrix (GLSZM), and neighborhood gray level different matrix (NGTDM). All radiomics features were standardized by Z-score transformation to strengthen the data comparability and reduce bias. We only included features with a good agreement (ICC > 0.75). The univariate logistic analysis was performed after the results were obtained to include the features with p < 0.10 for further study. Subsequently, the least absolute shrinkage and operator (LASSO) method was used to select radiomics features with excellent predictive properties.

### 2.4 Establishment of models and performance evaluation

#### 2.4.1 CAD model

S-Detect is a more interactive CAD system based on a specific deep learning algorithm: a convolutional neural network. Deep learning is an intricate multi-layer neural network architecture consisting of input, hidden, and output layers. S-Detect can realize precise decisions and identify benign and malignant nodules by learning a large amount of training data, extracting high-order statistics, and optimizing the balance of input and output data through many hidden layers (15, 16).

#### 2.4.2 Radiomics model

The Rad-Score (radiomics score) for each lesion was computed based on the estimated weighting coefficient of the selected features on each transverse and longitudinal section. Then, the radiomics model was ultimately constructed using this Rad-Score. Moreover, the nomogram was developed by radiomics labels to quantify the possibility of malignancy risk and evaluate high- and low-grade thyroid nodules.

We used the area under the receiver operating characteristic curves (AUCs) and calibration curves to evaluate the performance among the models and the senior radiologist in discriminating between benign and malignant nodules.

### 2.5 Optimizing the ACR TI-RADS using the models

Based on the nodule’s level and maximum diameter, ACR TI-RADS offers three recommendations: no biopsy, US follow-up, or biopsy (4). Both models had binary outputs of high and low malignant risks, and the results were used to upgrade or downgrade ACR TI-RADS management recommendations to explore the possibility of reducing unnecessary biopsies. More specifically, if our assessment indicated a high risk of malignancy, an upgrade was performed, such as no biopsy to follow-up or follow-up to FNA, or FNA remained unchanged; otherwise, when nodules were classified as low risk, we downgraded recommendations. Ultimately, we compared the diagnostic performance of the new risk stratification model with the original ACR TI-RADS recommendations.

### 2.6 Statistical analysis

The continuous variables were described with the median (interquartile range), and categorical variables were presented as frequencies or percentages. The Student’s t-test, chi-square test, and Fisher’s exact test were used for the univariate statistical analysis, as appropriate. The AUCs with 95% confidence intervals (CIs) were calculated to assess model performance for classifying benign and malignant thyroid nodules. DeLong’s test was employed to analyze between-group differences. Model calibration performance was assessed using calibration curves.

Additionally, the diagnostic value of the management recommendation was evaluated by calculating sensitivity, specificity, positive predictive value (PPV), negative predictive value (NPV), accuracy, and unnecessary FNA rates (no biopsy and follow-up were considered negative, and the biopsy was positive). We applied the maximum Youden index (sensitivity + specificity − 1) as the optimal cutoff value of the radiomics model to dichotomize all nodules into two groups (high and low risk of malignancy, similar to the CAD model) for discussing potential complementary roles to the ACR guidelines.

Statistical analyses were conducted using the SPSS for Windows version 25.0 (IBM Corporation) and R statistical software version 4.1.30 (R Foundation for Statistical Computing; https://r-project.org). A two-tailed p value < 0.05 was regarded as statistically significant.

## 3 Results

### 3.1 Study population

Of the 262 thyroid nodules with complete imaging data and confirmed pathological diagnoses from 148 unique patients (median age, 43 years, 202 women), 157 (59.9%) were malignant, while the remaining 105 (40.1%) were benign (**Table 1**). Patients of the malignant group were younger and malignant nodules were significantly smaller than benign nodules (p < 0.001). There were statistical differences in the ACR TI-RADS level in this cohort. (p < 0.001).

**Table 1:** Patient demographics and nodule characteristics (stratified by pathologic diagnosis).

Variables	All Nodules (n = 262)	Benign (n = 105)	Malignant (n = 157)	p value
Age (y)	43 (34, 53)	52 (38, 58)	38 (33, 49)	<0.001
Sex	202/60 (77.1/22.9)	83/22 (79.0/21.0)	119/38 (75.8/24.2)	0.643
Location				0.545
Left	126 (48.1)	52 (49.5)	74 (47.1)	
Right	124 (47.3)	50 (47.6)	74 (47.1)	
Isthmus	12 (4.6)	3 (2.9)	9 (5.7)	
Nodule size (mm)	10.0 (6.2, 18.9)	14.7 (7.1, 30.8)	9.0 (5.7, 12.7)	<0.001
ACR TI-RADS level				<0.001
TR1	4 (1.5)	4 (3.8)	0 (0.0)	
TR2	31 (11.8)	30 (28.6)	1 (0.6)	
TR3	14 (5.3)	11 (10.5)	3 (1.9)	
TR4	78 (29.8)	47 (44.8)	31 (19.7)	
TR5	135 (51.5)	13 (12.4)	122 (77.7)	

Data are presented as medians with interquartile ranges in parentheses or number parentheses are persentages. ACR TI-RADS, American College of Radiology Thyroid Imaging Reporting and Data System.

### 3.2 Overall diagnostic performance of the models

**Figure 3:**
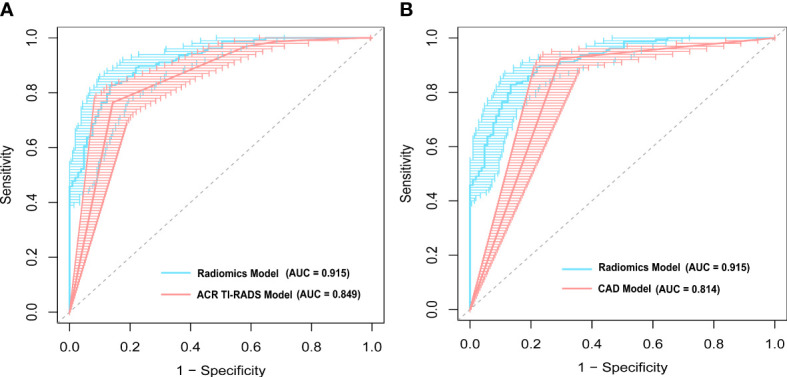
A comparison of receiver operating characteristic (ROC) curves between the radiomics model and **(A)** the American College of Radiology Thyroid Imaging Reporting and Data Systems (ACR TI-RADS) model, and **(B)** computer-aided diagnosis (CAD) model. The area under the ROC curve (AUC) was 0.915 for the radiomics model, which was significantly higher than that of the ACR TI-RADS (p = 0.004) and CAD models (p < 0.001).

**Figure 3** demonstrates the receiver operating characteristic (ROC) curves of three models for discriminating malignant and benign nodules. The AUCs of the radiomics, ACR TI-RADS, and CAD models were 0.915 (95% confidence interval (CI): 0.881–0.947), 0.849 (95% CI: 0.804–0.894), and 0.814 (95% CI: 0.766–0.863), respectively, as shown in **Table 2**. Compared with the senior radiologist, the CAD model had a higher sensitivity, and our radiomics model tended towards a higher AUC. The comparative results showed that the radiomics model yielded a higher performance than the ACR TI-RADS and CAD models, and DeLong’s test showed that the differences were statistically significant (p = 0.004, p < 0.001, respectively).

**Table 2:** Diagnostic performances comparison of the radiomics, CAD, and ACR TI-RADS models for thyroiuds nodules.

Model	AUC (95%Cl)	Sensitivity (%)	Specificity (%)	Accuracy (%)	PPV (%)	NPV (%)	p value*
Radiomics	0.915 (0.881-0.947)	83.4 (131/157)	86.7 (91/105)	84.7 (222/262)	90.3 (131/145)	77.8 (91/117)	NA
CAD	0.814 (0.766-0.863)	92,4 (145/157)	70.5 (74/105)	83.6 (219/262)	82.4 (145/176)	86.0 (74/86)	<0.001
ACR TI-RADS	0.849 (0.804-0.894)	77.7 (122/157)	87.6 (92/105)	81.7 (214/262)	90.4 (122/135)	72.4 (92/127)	0.004

Unless otherwise specified, data are presented as AUCs with 95% Cis in brackets, and data are percentages with numerators/denominator in parentheses. CAD, computer-aided diagnosis; ACR TI-RADS, American College of Radiology Thyroid Imaging Reporting and Data System; AUC, area under the receiver operating characteristic curve; CI, confidence interval; PPV, positive predictive value; NPV, negatie predictive value; NA, not applicable. *p values reflect the diagnostic efficacy AUC of each model compared to the radiomics model. DeLong’s test was used for statistical analysis.

**Figure 4:**
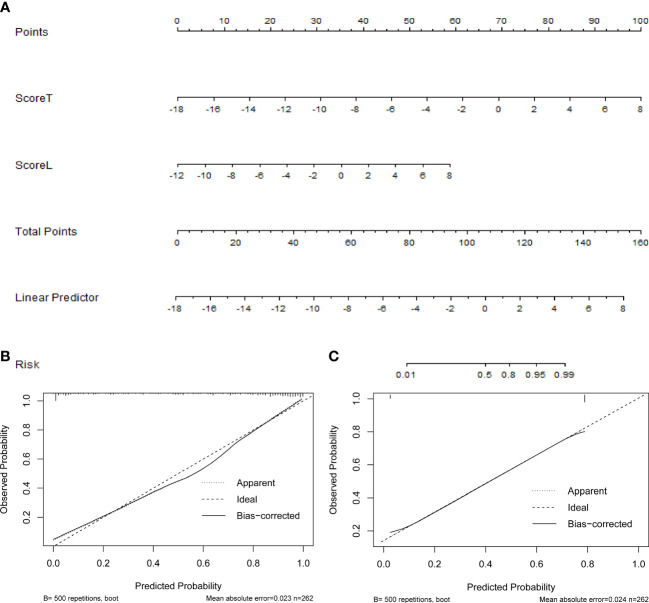
**(A)** The nomogram based on the radiomics model, the calibration curves of **(B)** the radiomics, and **(C)** computer-aided diagnosis (CAD) models.

In the radiomics model (**Supplementary Material**), the two variables, transverse and longitudinal radiomics label scores of thyroid nodules, that were statistically significant in the univariate statistical tests were entered into the model, and then applied to construct the nomogram (**Figure 4A**). In this visualization, each nodule could obtain predicted risk values for thyroid nodules by summing the scores for each variable. According to the ROC curve, the optimal cutoff value for the “risk of malignant nodules” was 0.656.

**Figures 4B, C** show the calibration curves of the radiomics and CAD models for predicting thyroid nodules, which illustrates that both models have good agreement between the observed and predicted values.

### 3.3 The role of management recommendations

The original ACR TI-RADS management recommendations categorized 87 nodules as FNA, and 58 of them were malignant. When both models were applied to revise the ACR TI-RADS, our risk stratifications significantly improved the performance. Specifically, the CAD model resulted in the downgrading of 54 nodules (29 from biopsy to follow-up), whereas 118 were upgraded (116 from follow-up to biopsy), and the radiomics model resulted in the downgrading of 83 nodules (39 from biopsy to follow-up), whereas the reassigning from biopsy to follow-up occurred for 97 nodules (**Table 3**). However, 12 malignant thyroid nodules were missed in the revised CAD model, and 26 tumors were missed in the revised radiomic model. **Table 4** shows the diagnostic performance of the original ACR TI-RADS and our revised risk stratification system. Compared with the ACR guidelines, both revised CAD and radiomics recommendations have impressive diagnostic performance, such as higher sensitivity, accuracy, PPV, and NPV, and decreased unnecessary FNA rates. In addition, the improvement scale of the radiomics model in the unnecessary FNA rates was more pronounced (33.3–16.7% vs. 33.3–9.7%). From the perspective of reducing missed diagnoses, the CAD model combined with TI-RADS is more effective.

**Table 3:** Distribution of ACR TI-RADS guidelines revised by our models.

Histopathology	Original ACR TI-RADS			Revised by CAD model			Revised by radiomics model			
	No Biopsy	Follow-up	Biopsy	No Biopsy	Follow-up	Biopsy	No Biopsy	Follow-up	Biopsy	Total
Benign	34	42	29	51	25	29	63	28	14	105
Malignant	1	98	58	6	6	145	15	11	131	157
Total	35	140	87	57	31	174	78	39	145	262

Data are presented as numbers of nodules. ACR TI-Rads, American College of Radiology Thyroid Imaging Reporting and Data System; CAD computer-aided diagnosis.

**Table 4:** The effects compared among original ACR TI-RADS management recommendations and revised diagnoses based on our models.

Model	Sensitivity (%)	Specificity (%)	Accuracy (%)	PPV (%)	NPV (%)	Unnecessary FNA rate (%)
Original ACR TI-RADS	36.9 (56/157)	72.4 (76/105)	51.1 (134/262)	66.7 (58/87)	43.4 (76/175)	33.3 (29/87)
Revised by CAD model	92.4 (145/157)	72.4 (76/105)	84.4 (221/262)	83.3 (145/174)	86.4 (76/88)	16.7 (29/174)
Revised by radiomics model	83.4 (131/157)	86.7 (91/105)	84.7 (222/262)	90.3 (131/145)	77.8 (91/117)	9.7 (14/145)

Unless otherwise specified, data percentages with numerator/denominator in parentheses. ACR TI-RADS, American College of Radiology Thyroid Imaging Reporting and Data System; CAD, computer-aided diagnosis; PPV, positive predictive value; NPV, negatie predictive value, FNA, fine-needle aspiration.

## 4 Discussion

In this study, we found that the radiomics model presented with a significantly higher diagnostic accuracy for predicting the malignancy risk of thyroid nodules compared with the CAD model (p < 0.001) and a senior radiologist (p = 0.004), while the CAD model showed a higher sensitivity (92.4 vs. 83.4, 77.7%). In addition, we applied our systems to revise the ACR TI-RADS management recommendations, especially the radiomics model, successfully optimizing its performance and reducing unnecessary biopsies.

Our study had some unique characteristics. First, in contrast to most previous studies using the retrospective radiomics strategy, we adopted a prospective design process in which a senior radiologist acquired images and applied strict quality control, thus making all high-quality images more standardized. Second, most S-detect-related studies developed models solely on the transverse section of the lesion for analysis (8, 9, 11, 12). The largest segment (longitudinal section) and its vertical section (transverse section) were chosen in our study to increase the lesion characteristics and reduce the impact of subjective factors. Additionally, the radiomics features of a thyroid nodule were separately extracted from two objective vertical US images, which may provide more detailed information and reflect the tumor heterogeneity.

Radiomics is widely recognized as an important method for medical image analysis in oncology research (17). In this study, we developed a radiomics model for the differentiation of thyroid nodules and constructed a nomogram using the radiomics label. Our model was established through the logistic regression approach, which is the most commonly used supervised learning model in US radiomics (18). The application of radiomics showed adequate diagnostic performance in predicting the malignancy of thyroid nodules with an AUC of 0.915, which was consistent with previously reported studies (19–21). Several studies have reported that the CAD system is a promising approach for solving practical difficulties in clinical diagnosis (8–12, 22). Eun et al. (23) reported a high sensitivity of up to 88.6% and suggested that the CAD system could be useful as decision-making support to rule out cancer. In this study, we also found that the CAD system had a high sensitivity (92.4%) and accuracy (83.6%). The thyroid CAD system used in this study was integrated into the US system, which enabled the use of CAD system in real-time clinical practice. Furthermore, a real-time second opinion on the decision for the necessity of FNA is possible with the present system. Due to its simplicity and reduced analysis time, this system would be simpler to apply in routine practice (8). Therefore, we concluded that the CAD system could reduce the time required for the interpretation process of thyroid nodules and diagnose them as benign or malignant, making it a simple system to screen thyroid nodules for high sensitivity. The radiomics and CAD models constructed in our study showed good robustness and also illustrated the strong generalization ability of our method.

The ACR TI-RADS is based on an expert consensus, literature review, and partial analysis of the database of proven nodules; its core objective is to focus on clinically significant thyroid cancers and reduce the FNA of benign nodules (4). Wildman et al. (7) used genetic algorithms to improve the performance of artificial intelligence TI-RADS by optimization of the points assigned to each TI-RADS feature, which can validate the ACR TI-RADS while improving specificity and maintaining sensitivity. In our study, we attempted to explore the potential complementary role of radiomics and CAD models to ACR TI-RADS Risk Stratification for thyroid nodule management; the results showed that both models could provide additional gains in performance, especially in terms of sensitivity and accuracy (**Table 4**). Notably, both revised models successfully reduced unnecessary biopsies compared with the ACR TI-RADS, especially the radiomics model. This may support that the radiomics strategy can capture information that is beyond visual interpretation and interpret heterogeneity within lesions. Using quantitative information on radiomics features could be more effective as a complementary tool to management recommendations. On the other hand, although the S-detect model is based on a deep learning algorithm generated using a large database, the algorithm relies on the quality of the annotated US image features, which will inevitably depend on the reader’s experience. In addition, the deep learning method may suffer from possible over-fitting. In summary, we recommended that radiologists appropriately optimize the ACR TI-RADS risk stratification system with the assistance of new technologies.

Our study had several limitations. First, this study did not include any large-scale test datasets to validate. Thus, it will be necessary to conduct a more stringent internal and external validation with a larger sample size representing the screening population. Second, this study only used static vertical section images. In future studies, model evaluation using cine clips that include the entire thyroid nodule and surrounding thyroid parenchyma may be necessary to avoid losing the risk features for malignancy. Third, thyroid nodules have various histological subtypes with different molecular mechanisms, grades of malignancy, clinical aggressiveness, and imaging appearances (24). The low occurrence rate of non-papillary carcinoma determines a relatively low percentage in our study. Future efforts will be warranted to include a larger sample size with varied pathological types to further enhance the generalizability and performance.

In conclusion, our study provides evidence that the radiomics strategy and CAD system both have the potential to predict malignancy in thyroid nodules and suggests a simple method to optimize the ACR TI-RADS recommendation. This approach finds the potential complementary roles of both models to the guidelines, which can more precisely help in the classification of thyroid nodules and successfully reduce unnecessary biopsies, especially the radiomics model, which is recommended due to its lower unnecessary FNA rates.

## Data availability statement

The raw data supporting the conclusions of this article will be made available by the authors, without undue reservation.

## Ethics statement

The studies involving human participants were reviewed and approved by the ethical review committee of the Third Xiangya Hospital of Central South University. Written informed consent to participate in this study was provided by the participants’ legal guardian/next of kin. Written informed consent was obtained from the individual(s), and minor(s)’ legal guardian/next of kin, for the publication of any potentially identifiable images or data included in this article.

## Author contributions

MX designed the research, analyzed, and drafting of the manuscript. FS contributed to the analysis and provided statistical advice for this manuscript. PZ collected data and reviewed the manuscript. YZ and YW analyzed data. YX provided instructive advice and supervision. All authors contributed to the article and approved the submitted version.
