# Supplementary material for: Ultrasonography-based radiomics and computer-aided diagnosis in thyroid nodule management: performance comparison and clinical strategy optimization
Source: Front Endocrinol (Lausanne). 2023 May 12;14:1140816. doi: 10.3389/fendo.2023.1140816 (PMC10213653; doi:10.3389/fendo.2023.1140816)
Supplement: Supplementary file 1 [file Table_1.docx]

Supplementary Material

Ultrasonography-Based Radiomics and Computer-Aided Diagnosis in Thyroid Nodule Management: Performance Comparison and Clinical Strategy Optimization

Mengwen Xia^1^, Fulong Song^2^, Yongfeng Zhao^1^, Yongzhi Xie^2^, Yafei Wen^1^, Ping Zhou^1*^

*** Correspondence:** Ping Zhou: zhouping1000@hotmail.com

# Supplementary Data

## Radiomics model

Calculation formulas for the Radiomics score are shown below:

Radiomics score_ longitudinal

=-2.228 + X10Percentile×-1.873 + DifferenceEntropy.1x-3.618

+ Maximum.2x-4.496 + Imc1.2x3.671 + ZoneEntropy.2x3.144 + Maximum.4x1.639

+ Skewness.4x-0.880 + RunEntropy.4x11.604 + Median.6x0.174 + Skewness.7x-0.356

+ LongRunHighGrayLevelEmphasis.8x-2.444

+ SizeZoneNonUniformity.8x-0.847 + Complexity.8x1.257

Radiomics score_ transverse

= 13.103+ Rangex5.436 + Busyness.1x-1.354 + LowGrayLevelZoneEmphasisx1.740

+ Minimum.1x1.230 + ZoneEntropy.1x-1.590 + ClusterProminence.2x-6.890

+ Maximum.3x-2.135 + Minimum.4x-3.137

+ HighGrayLevelZoneEmphasis.6x-3.992 + Maximum.7x-2.214

+ X10Percentile.8x-0.862+ LongRunHighGrayLevelEmphasis.8x-3.128

+ SmallAreaHighGrayLevelEmphasis.8x0.610 + Contrast.17x-1.890

Radiomics score

=-0.200 + 0.534xscore_ longitudinal + 0.702xscore_ transverse
